# Beyond Vaccination: Exploring Young Adults’ Awareness, Knowledge, and Attitudes Related to Sexually Transmitted Infections in Romania

**DOI:** 10.3390/vaccines13030322

**Published:** 2025-03-18

**Authors:** Alexandra-Ioana Roșioară, Bogdana Adriana Năsui, Nina Ciuciuc, Dana Manuela Sîrbu, Daniela Curșeu, Romulus Florian Oprica, Codruța Alina Popescu, Rodica Ana Ungur, Tamara Cheșcheș, Monica Popa

**Affiliations:** 1 Department of Community Medicine, Iuliu Hațieganu University of Medicine and Pharmacy, 400349 Cluj-Napoca, Romania; alexandra.rosioara@umfcluj.ro (A.-I.R.); nina.ciuciuc@umfcluj.ro (N.C.); dsirbu@umfcluj.ro (D.M.S.); dcurseu@umfcluj.ro (D.C.); monica.popa@umfcluj.ro (M.P.); 2 Research Center in Preventive Medicine, Health Promotion and Sustainable Development, Iuliu Hațieganu University of Medicine and Pharmacy, 400349 Cluj-Napoca, Romania; 3 BrandBerry Research Institute, 500461 Brașov, Romania; ro@romulusoprica.ro; 4 Department of Abilities Human Sciences, Iuliu Hațieganu University of Medicine and Pharmacy, 400012 Cluj-Napoca, Romania; cpopescu@umfcluj.ro; 5 Department of Medical Specialties, Faculty of Medicine, Iuliu Hațieganu University of Medicine and Pharmacy, 400012 Cluj-Napoca, Romania; ungurmed@yahoo.com; 6 Department of Medical Psychology and Medical Communication, Faculty of Medicine, University of Medicine and Pharmacy “Carol Davila”, 050474 Bucharest, Romania; tamara.chesches@rez.umfcd.ro

**Keywords:** sexually transmitted infections, young people, sexual education, risk perception, health communication, vaccine literacy, health literacy, social determinants, public health

## Abstract

Background and Objectives: Romania has the highest rate of cervical cancer in Europe. The aim of this study is to measure the level of sexual health knowledge among participants and determine the extent to which factors such as age, gender, education level, access to sexual health resources, and cultural background influence their knowledge. Materials and Methods: A cross-sectional study was conducted on 1089 Romanian youth participants aged 18–35 years. A self-administered online questionnaire was used concerning the level of knowledge relating to STIs, contraception methods, and preventive attitudes during the 2023–2024 academic year. Results: Most of the participants (93,8%) scored a “good-to-excellent” STI level of knowledge. Despite this, 71.9% of the responders had never taken an HIV test, and 63.5% had never been tested for other STIs. Logistic regression analysis revealed a direct association between higher STI knowledge levels among respondents with age (*p* < 0.001), underage sexual debuts (*p* = 0.018), greater parental education (*p* = 0.016), and those who studied health sciences (*p* < 0.001). Conclusions: This study highlights the critical need for health communication campaigns to enhance STI knowledge and vaccine literacy to improve the vaccination rates among young people in Romania. The identified knowledge gaps, frequent misconceptions, and barriers to STI testing underscore the importance of comprehensive sexual health education, public health initiatives for reducing the stigma associated with STIs, and improved access to healthcare services for young people.

## 1. Introduction

The World Health Organization (WHO) defines sexual health as a multifaceted state of well-being (physical, emotional, mental, and social) related to sexuality, emphasizing positive experiences free from coercion, discrimination, or violence [1].

Sexually transmitted infections (STIs) pose a significant global public health burden, particularly among young people. Estimates suggest that nearly half of the new cases of STIs occur in 15–24-year-olds, with 1 in 20 teenagers acquiring a bacterial STI annually [2]. STIs have a profound impact on sexual and reproductive health worldwide. More than 1 million curable STIs are acquired every day worldwide in people aged 15–49 years old, the majority of which are asymptomatic [3].

In a series of reports released by the European Centre for Disease Prevention and Control (ECDC), a concerning rise in the number of STIs across Europe was revealed, indicating troubling trends and significant public health implications [4], and the same has been observed in the United States [5]. Romania faces significant challenges in addressing sexually transmitted infections (STIs), particularly among adolescents. While most STIs are curable, their early detection and treatment are crucial to prevent complications. The lack of national STI screening programs in Romania hinders both their effective treatment and the collection of essential STI data [6]. In addition, Romania bears a disproportionate burden of sexually transmitted human papillomavirus (HPV)-related cervical cancer, accounting for 7.5% of yearly cases in Europe and experiencing a mortality rate of 14.2 per 100,000 women, which is triple the European Union average [7,8,9]. In Romania, 22.5% of teenagers have their first sexual contact before the age of 15, and 11.8% have their first birth before this age [10].

In Romania, the subject of sexual education is included in the discipline “health education”, which is not mandatory in schools; it is an optional discipline which may be included as part of the curriculum at a school’s discretion [11]. A report about this program was created by Save the Children Romania two years ago, showing that only 6% of students have access to health education [12]. A 2023 healthcare reform law which was passed in Romania outlines how health education, including sexual health, can be implemented with support from various healthcare professionals and organizations [13]. It was reported that health education will be mandatory, and curriculum development will be overseen by the Ministers of Health and Education. However, the guidelines and implementation strategy are not yet available. Unstandardized educational efforts such as campaigns or different programs do exist in schools. Health education and healthy lifestyle promotion are crucial for the well-being of Romanian youths [14]. Successful national programs require sustained campaigns, continuous funding, streamlined bureaucracy, and gender-neutral human papillomavirus (HPV) vaccination [15]. A gender-neutral campaign also favors the development of a herd immunization effect among adolescents and young people, which will have beneficial results [16].

The definition of “youth” varies across international and national contexts. While the United Nations defines “youths” as individuals aged 15–24 for statistical purposes [17], the European Commission extends this range to 15–29 years [18]. In Romania, the Youth Law (Law no. 350/2006, updated in 2024) defines youths as citizens aged 14–35, although this is subject to potential future revisions [19].

Knowledge gaps regarding HPV and vaccination persist despite health education efforts, necessitating continued healthcare provider training [20]. Limited access to comprehensive sexual education, particularly in rural areas, contributes to high teenage pregnancy rates in Romania, emphasizing the need for multi-faceted interventions [21]. While the ideal approach to adolescent sexuality education remains debated, comprehensive programs, whether school-based or with parental involvement, are crucial for providing complete and culturally sensitive information [22].

Health literacy (HL) is a concept defined as “the cognitive and social skills which determine the motivation and ability of individuals to gain access to, understand and use information in ways which promote and maintain good health” [23]. For instance, Thompson et al. demonstrated a positive correlation between higher levels of health literacy (HL) and desirable health behaviors, such as regular Pap tests among those aware of HPV as a health risk [24]. Conversely, a large European study (n = 8000) revealed inadequate health literacy levels, especially within vulnerable categories such as people with poor health status, low socioeconomic status, and lower education levels, older ages, as well as those who report high usage of healthcare services [25]. According to Becker’s health belief model, people’s knowledge and attitudes toward a health-related problem might correlate with their future behavior [26].

People at risk of STIs tend to minimize this risk and have average health literacy. A study from 2024 showed that many patients use online resources as a source of information in cases of health problems [27]. While online health resources offer benefits, self-diagnosis via the internet can lead to unwarranted health anxiety in the absence of professional medical follow-up [28]. There is a tendency for young people to underreport their risk of contracting STIs, as most of them do not think that their current sexual behavior can put them at risk of infection [29].

Young people should be at the center of strategies to control STIs and HIV infection, and addressing inaccurate perceptions of risk may be key to improving safer sexual practices [30]. A study from Italy, which was conducted in 2021, involved students attending non-biomedical high schools and university faculties. The effectiveness of an educational intervention for improving the limited baseline knowledge of HIV and STIs was reported, particularly within this category [31]. Another study from the USA which investigated levels of awareness and knowledge of HPV and the HPV vaccine among university students also showed that a relatively high level of awareness concerning HPV was observed. The gaps in knowledge suggest that further efforts are necessary to educate young adults [32].

Making the HPV vaccine part of the National Vaccination Program in Romania would significantly improve access to this important preventive measure for the general population. Cost and lack of information were identified as key barriers to HPV vaccination among parents who had not vaccinated their children [33].

This study holds significant importance due to the scarcity of research on sexual health knowledge, attitudes, and behaviors among young people in Romania, particularly in the absence of a comprehensive national sexual education program within the mandatory school curriculum. This research contributes valuable insights to a critical yet under-researched area, highlighting the need for evidence-based interventions and policy development to improve sexual health outcomes for Romanian youth.

The aim of this study is to measure the level of sexual health knowledge among participants and determine the extent to which factors such as age, gender, education level, access to sexual health resources, and cultural background influence their knowledge.

## 2. Materials and Methods

### 2.1. Study Design and Participants

We ran a cross-sectional study which was conducted based on an online questionnaire specifically designed by the authors for this study. It was based on data from the literature [2,31,34,35,36,37,38] relating to the level of STI-related knowledge, preferred sources of information, use of contraception, and sexual behaviors. It was distributed online via Google Forms^®^ (Google LLC, web-based, Mountain View, CA, USA) to young people who were enrolled to study biomedical and non-medical courses at Romanian universities. The participants were randomly selected from the nationwide population across various regions of the country; the majority were from the northwestern part, which is the larger part of the country, as well as from the western or southern parts of Romania, which includes the capital. This study was performed during the 2023–2024 academic year.

For the inclusion criteria, eligible participants were young people from Romania aged 18–35 years, who were able to complete the questionnaire, enrolled at university (or graduated students), were raised in Romania, and had access to the internet. (The questionnaire was online on the Google Form platform).

For the exclusion criteria, responders <18 and >35 years of age without consent for completion of the questionnaire or who submitted incomplete questionnaires were excluded from this study.

A pilot test of the questionnaire was performed on 30 students aged 19–23 years to evaluate its reliability. Spearman’s correlation coefficient was used to assess its reliability (r = 0.763).

In Romania, there are 4,195,831 young people between the ages of 18 and 35, according to Romanian statistical reports. Out of them, 538,700 were enrolled in university [39]. We calculated the sample size needed for our study using Paniott’s formula; for a 95% confidence level and a 5% margin of error, the required sample size was 384. However, we included 1089 participants to increase the significance power of this study. This study originally included 1104 respondents, but all of the questionnaires which were out of the specified age range, completed incorrectly, or contained incomplete data were eliminated from the study and were not included in the statistical analysis.

### 2.2. Data Collection and Questionnaire

Responders’ participation in our research was voluntary and not a condition for completing a university course. The respondents received no compensation. Volunteering responders received a link to the questionnaire and filled it in anonymously. On average, the time taken to complete the questionnaire was 15 min. By answering the questions, they consented to participate in the study.

This study employed a mixed-methods recruitment strategy to maximize participation, including in-person surveys during university lectures, online dissemination via Google Forms, social media, and email, as well as collaboration with a medical doctor and influencer to reach their followers (participants >35 years old were excluded). Concerning this recruiting strategy, which used mixed methods, while it is impossible to completely guarantee that no individual completed the survey multiple times, several measures were taken to minimize this risk. A significant portion (55%) of the responses was collected in a controlled classroom setting where students completed the survey under the direct supervision of their instructors, reducing the likelihood of duplicate submissions within this group. The survey’s introduction clearly emphasized the importance of honest and single participation, appealing to the ethical responsibility of the respondents. While the possibility of some duplicate entries cannot be entirely ruled out, it is believed that their number would be minimal and unlikely to significantly impact the overall findings of this study.

The questionnaire consisted of 41 questions and was organized into two sections: (A) background and context, consisting of (a) demographic data (age, gender, area of living, ethnicity, and religion); (b) living situation (upbringing and current living conditions); and (c) personal and parental education level and field of study, and (B) sexual health and lifestyle, consisting of (a) STI awareness (self-assessment of STI knowledge level, perceived value of STI info, risk perception, self-perception of unprotected sex risks, and preferred sources of information); (b) STI knowledge level assessment (specific questions on transmission, prevention, and consequences (presented in Supplementary Table S1)); (c) sexual history (relationship status, sexual debut, partner demographics, sexual orientation, recent partners); and (d) STI prevention attitudes (preferred methods of contraception, reasons for no contraception, contraception testing, HIV testing history, and STI testing history).

The questionnaire was in Romanian, as alternative versions in languages other than Romanian were considered unnecessary.

A comprehensive scoring system was meticulously developed for questions 19, 20, 21, 22, 23, 24, 27, 28, 29, 31, and 34. Each correct response was awarded a value of 1. In instances where multiple correct answers were possible, the 1 point was equitably distributed among the correct options. Conversely, incorrect responses were penalized with a value of −1. For questions with multiple incorrect options, the −1 point was similarly distributed among those options. The final score for each participant was calculated by summing the values obtained for all answered questions. The interval of scoring was set between −11 and 11. This scoring methodology resulted in a score range from −6.25 to 11, providing a nuanced assessment of participant performance. For the level of STI knowledge, we calculated scores for the responders to summarize all the questions they responded to in order to place them into level of knowledge categories (very poor, poor, medium, good, very good, and excellent). In order to simplify data reporting and analysis, we proceeded to dichotomize knowledge status into high versus low levels (i.e., poor to average versus good to excellent). In Supplementary Table S1, the questions and coding for the included variables are presented.

### 2.3. Ethical Considerations

This study was conducted according to the guidelines of the Declaration of Helsinki and was approved by the Ethics Committee of the Cluj Napoca University of Medicine and Pharmacy (No. 111/4 June 2024). Additionally, all of the participants were fully briefed about the aim of the study, the data collection, and the fact that it was anonymous. They gave their informed consent to participate in the study by putting a check mark at the beginning of the form. The influencer was fully briefed on the study’s purpose and provided informed consent for their involvement.

### 2.4. Statistical Analyses

All statistical analyses were conducted using IBM SPSS Statistics (version 25, IBM Corp., Armonk, NY, USA) and Microsoft Excel (Microsoft Office 2010, Albuquerque, NM, USA). Descriptive and inferential analyses were performed to address the study’s research questions regarding young people’s level of knowledge and awareness as well as their determinants. Continuous variables (age) were summarized using means, standard deviations, and 95% confidence intervals. Categorical variables (gender, background environment, and parent education) were described using frequencies and percentages. To explore differences in the level of knowledge and outcomes between boys and girls or their backgrounds religions, or ethnicities, chi-squared tests were employed for the categorical variables. The relationships between key lifestyle factors (e.g., sexual history, STI awareness, and prevention attitudes) and level of STI knowledge (calculated score) were tested using chi-squared tests and cross-tabulations to identify statistically significant associations.

To identify independent predictors of young people’s level of knowledge, a multivariate logistic regression model was employed. The dependent variable was the calculated level of knowledge score (poor to average versus good to excellent), and the variables which achieved significance in the univariate analysis included gender, age, field of study, parent education, and age of sexual debut.

Statistical significance was set at *p* < 0.05 for all tests.

## 3. Results

### 3.1. Background and Context

The sample size was 1089 responders who were young people aged between 18 and 35 years with an average and standard deviation of 26 ± 2.85 years of age; who were undergraduate university students or postgraduate non-students; and who were raised in Romania. Regarding sex, we had 865 women (79.4%), 221 males (20.3%), and 3 participants who declared another gender (0.3%) in this study. Among them, 825 were studying health sciences (75.8%), and 264 were studying non-health subjects (24.2%). The study population included individuals from both rural areas (210 (19.3%)) and urban areas (879 (80.7%)). From the point of view of ethnicity, out of all of the responders, 1033 (94.9%) were of Romanian ethnicity, 3 (0.3%) were of Roma ethnicity, 33 (3.1%) were of Hungarian ethnicity, and 20 (1.8%) were of other ethnicities (Italian, Jordanian, or Lipovac). Regarding religion, there were 914 (83.9%) Orthodox individuals, 56 (5.1%) were Catholic, 35 (3.3%) were Protestants, 41 (3.8%) declared belonging to no religion, 11 (1.0%) were Baptists, 1 (0.1%) and 31 (2.8%) practiced other religions (undeclared). Table 1 shows the sociodemographic characteristics of the responders by number and percentage.

This study conducted a descriptive analysis regarding demographics, exploring the association between it and the level of knowledge for the calculated scores. As seen in Table 2, our results show that overall, the participants had good-to-excellent knowledge of sexual education (94.6%). Males were significantly more likely to have poor-to-average knowledge compared with females (*p* < 0.002). Younger participants (18–20) were more likely to have poor-to-average knowledge compared with the older age groups (*p* < 0.001). Urban residents were more likely to have good-to-excellent knowledge compared with rural residents (*p* = 0.081). A Roma ethnicity was associated with significantly lower levels of knowledge compared with other ethnicities (*p* = 0.022). Participants with both parents receiving higher education were more likely to have good-to-excellent knowledge (*p* < 0.001). Students were more likely to have good-to-excellent knowledge compared with those who were not (*p* < 0.001). Those studying health sciences were more likely to have good-to-excellent knowledge compared with those studying non-health sciences (*p* < 0.001).

### 3.2. Sexual Health and History

The study analyzed various factors relating to sexual health and their potential association with the level of STI knowledge among the participants. The results (Table 3) show that the majority of the participants identified as heterosexual (89.1%), but those identifying as “Other” had a slightly higher proportion with “poor-to-average” sexuality education levels compared with heterosexuals (*p* < 0.001). Participants who first had sexual intercourse at a younger age (<18) tended to have a higher proportion with “poor-to-average” sexuality education levels compared with those who started later. This trend was statistically significant (*p* < 0.001). Having a younger first partner was associated with a higher proportion of “poor-to-average” sexuality education levels (18.5%) and was statistically significant (*p* = 0.006). Participants with 1–2 recent sexual partners had a higher proportion with “good-to-excellent” sexuality education levels compared with those with no recent partners or 3–5 partners, demonstrating a statistically significant difference (*p* < 0.001). Participants with stable sexual partners had a higher proportion with “good-to-excellent” sexuality education levels compared with those without them (95.1% versus 91.4%) (*p* = 0.004).

### 3.3. STI Awareness and Risk Perception

The study evaluated the relationship between various factors related to self-perceived knowledge or attitudes toward STIs and the actual calculated STI knowledge levels among the participants. As seen in Table 4, there was a strong association between self-assessed knowledge and the calculated STI knowledge level (*p* < 0.001). Those who rated themselves higher tended to have better actual knowledge. The vast majority had a positive perception of STI information. Interestingly, those with a negative perception had a higher proportion of “poor-to-average” actual knowledge (57%), and this difference was statistically significant (*p* < 0.001). Most participants perceived themselves to be at low risk for STIs. There was no significant difference in actual knowledge between those with low- and high-risk perception (*p* = 0.17). Most participants considered themselves “risk-aware” regarding unprotected sex. Those who were “risk-unaware” had a significantly higher proportion of “poor-to-average” actual knowledge (*p* < 0.001). Regarding sources of information, “health specialists” were the most preferred source, followed by media and community. There was no significant difference in actual knowledge based on preferred information sources (*p* = 0.93).

### 3.4. STI Prevention Attitudes

This study also explored the relationship between various factors related to STI testing history, contraceptive practices, attitudes toward contraception, and the calculated STI knowledge levels among the participants. As seen in Table 5, the participants who underwent HIV or STI testing had significantly higher “good-to-excellent” STI knowledge levels compared with those who had not (*p* < 0.001 for both). Condoms were the most preferred method, followed by oral contraceptives and the pullout method. There was a significant association between the preferred methods and STI knowledge level (*p* < 0.001); those who preferred condoms tended to have better knowledge levels. Regarding the attitude toward contraceptive methods, most participants used contraceptive methods or did not have sexual contact. Certain attitudes, like believing pleasure is greater without contraception or that their partner is responsible for contraception, were associated with lower STI knowledge levels (*p* < 0.001).

### 3.5. Factors Associated with Level of STI-Related Knowledge

We ran multiple linear regression to predict the level of STI-related knowledge of young people based on age, gender, background, field of study, ethnicity, religion, parental education, attitude toward contraception, age of onset of sexual life, and sexual orientation, as seen in Table 6. According to the results, higher parental education levels were significantly associated with an increased chance of respondents demonstrating “good-to-excellent” STI knowledge (*p* < 0.05). This finding aligns with the expectation that greater parental education may contribute to better sexual health knowledge in their children. Field of study (e.g., studying health sciences) emerged as a statistically significant predictor of the STI knowledge level (*p* < 0.05). An underage sexual debut was associated with a 54% increase in the rates of achieving “good-to-excellent” STI knowledge (*p* < 0.05).

## 4. Discussion

The aim of this study was to measure the level of sexual health knowledge among participants and determine the extent to which factors such as age, gender, education level, access to sexual health resources, and cultural background influence their knowledge.

The descriptive data illustrated that most of the students had good-to-excellent knowledge overall about STIs, which was also concluded in an older study conducted in Romania [2]. Despite limited research on sexual education in Romania, recent studies highlighted a growing awareness of HPV vaccination [40], which is particularly crucial given the country’s disproportionately high rates of HPV-related cervical cancer. Romania’s incidence of cervical cancer is 2.5 times that of the European average, with mortality exceeding the European average by over three times [7,8,9].

The limited research on sexual education in Romania has several significant implications, including knowledge gaps which make it difficult to assess the true state of sexual health knowledge, attitudes, and behaviors among young people and the development of effective and targeted sexual education programs [2]. Another implication of limited research is leading to missed opportunities for preventing negative sexual health outcomes, such as unintended pregnancies and STIs [41]. Without robust research to inform policy and program development, sexual education initiatives may be ineffective or even counterproductive [42]. Also, stigmas and misinformation can arise when young people rely on inaccurate or incomplete information from unreliable sources, perpetuating stigmas and potentially leading to harmful practices [43]. Limited research may fail to identify the specific needs and vulnerabilities of certain groups or those from marginalized communities, leading to inequitable access to sexual health information and services [44]. When individuals overestimate their STI knowledge, they may be less likely to seek out further information or participate in educational programs. This can hinder efforts to address knowledge gaps and promote informed decision making about sexual health.

This study found a strong correlation between the field of study and knowledge level among health science students, with medical students demonstrating greater knowledge. This aligns with previous research indicating improved HPV knowledge among medical students throughout their studies. However, while knowledge is a factor, coping strategies and health locus of control are stronger predictors of vaccination intent [45]. Furthermore, a global study including Romanian medical students revealed gaps in the knowledge regarding cervical cancer risk factors, emphasizing the need for targeted education in this area [46]. This study observed that STI knowledge increased with age among non-health science students. This aligns with research indicating that older adolescent girls demonstrate greater awareness of HPV vaccination [47] and that information on sexual health is often inadequately obtained from qualified sources [48]. While this study did not specifically explore the relationship between psychopathology and sexual health [49], access to sexual health services [50], or parental communication patterns [21], these factors warrant further investigation within the Romanian context to develop comprehensive sexual health interventions. If people believe they already possess sufficient knowledge, then they may be less receptive to educational messages or interventions. This can reduce the effectiveness of public health campaigns aimed at increasing awareness and promoting safer sexual practices. Overconfidence in one’s knowledge can lead to a false sense of security and increased risk-taking behaviors. This can contribute to higher rates of STIs and unintended pregnancies.

While our study did not explicitly examine the accuracy of self-perceived STI knowledge across different demographics, we can explore potential trends based on the data we collected. Younger participants (18–20 years old) were more likely to have lower STI knowledge scores. It is possible that this group might overestimate their knowledge due to a lack of awareness of the breadth and depth of information surrounding sexual health. Individuals from rural areas demonstrated slightly lower knowledge scores compared with their urban counterparts. Limited access to information and resources in rural areas could contribute to both less knowledge and a potential overestimation of said knowledge. The Roma ethnicity group showed significantly lower STI knowledge scores. Cultural factors and potential barriers to accessing information could lead to an overestimation of knowledge within this demographic. Medical students might tend to overestimate their level of knowledge due to their educational backgrounds and access to resources, possibly underestimating their knowledge needs, assuming they are already well informed. This is conducive with another Polish study which showed that despite the availability of information, knowledge gaps regarding HPV and its vaccination persist, even among medical students [51].

Regarding protection, our study results indicate that the vast majority of the participants used condoms, but when asked about other preventive attitudes (e.g., STI testing history), most of them responded by reporting that they had never been tested. Other studies indicate that limited knowledge about cervical cancer and preventive measures, coupled with financial and time constraints, contribute to low participation in HPV vaccination and screening programs in Romania [15,52]. Furthermore, research has highlighted that negligence, a lack of information, and the absence of perceived risk are the primary reasons for neglecting cervical cancer screening [53,54]. Misinformation can lead to a false sense of security or promote harmful practices, increasing the risk of STIs and unintended pregnancies. For example, believing myths about STI transmission or ineffective prevention methods can lead to unprotected sex and higher infection rates. These factors underscore the urgent need to improve the awareness of and accessibility to preventive services to address Romania’s significant cervical cancer burden. Research indicates that vaccination intentions among young adults are influenced by multiple factors, with predictive models explaining 51% and 60% of the variance in HPV and influenza vaccination intent, respectively [55]. Furthermore, HIV testing rates in Europe, including Romania, remain suboptimal, highlighting the need for improved access and awareness [56].

The present study revealed that the preferred sources of information were health specialists, while extremely few obtained information from the community (parents, teachers, and church). Concerning health communication, other research suggests that young people often initiate conversations about sexual and reproductive health (SRH) with a parent of the same gender, typically following significant life events [57]. However, intergenerational discrepancies exist in perceptions of sex education, with parents often underestimating its benefits [58]. This disconnect is exacerbated by the persistent taboo nature of sex education in Romanian society and the delegation of responsibility between families and schools [59,60]. While online platforms offer opportunities for parental support and information sharing, concerns remain regarding information quality [61]. The effectiveness of parental sexual education programs is influenced by a multitude of factors, necessitating further research and standardized evaluation tools to assess program quality and participant satisfaction [62,63]. Peer-to-peer and early SRH education appear to be promising avenues for enhancing program effectiveness and participant engagement [63].

There was a decreasing tendency in the incidence of syphilis and gonorrhea in adolescents aged 15–19 during the studied period [64]. Additionally, another study demonstrated that in the last 10 years, in Romania, the incidence of syphilis has had a downward trend, but with an increase in syphilis–HIV co-infection and neurosyphilis cases [65].

The study showed that most of the subjects had engaged in intercourse first at 17–18 years old (48.58%). The number of individuals who had started their sexual lives earlier than at 17 years of age was higher in males and in young subjects (*p* < 0.001) [66]. There is a continued need to provide health services to adolescents which include contraceptive choices and condoms and involve them in the design of services. Schools may be a good place in which to provide these services [67]. Despite limited research on audience evaluation, entertainment media’s scalability and cost-effectiveness make it a potential tool for promoting safer sex, particularly among large youth populations. The existing evidence base requires strengthening, highlighting the need for further research on the efficacy of entertainment education in promoting sexual health [68].

This study employed a mixed-methods approach to data collection, utilizing both direct online dissemination and influencer-promoted dissemination. Both methods proved effective, with direct dissemination offering unfiltered access and influencer collaboration extending the reach to potentially underserved populations [69,70]. This approach aligns with the increasing recognition of influencer engagement in public health initiatives [71,72], highlighting the potential for interdisciplinary collaboration between marketing and public health to address priority health concerns [73]. Furthermore, integrating gamification elements into sexual health education platforms shows promise for increasing engagement and promoting behavior change [74]. Campaigns should prioritize providing clear, accurate, and accessible information about STIs, as well as their transmission, prevention, and treatment. This information should be tailored to different audiences and disseminated through various channels, including social media, schools, and healthcare settings.

This study acknowledges several limitations inherent to its cross-sectional design. Firstly, while associations between the determinants and outcomes can be observed, this design can rule out the establishment of definitive causal relationships. Secondly, the study sample was predominantly female (79.4%), which may limit the general applicability of the findings to the broader population. While the overrepresentation of women in the sample may have influenced the study’s outcomes, it also offers valuable insights into the experiences and perspectives of this demographic. Women may be more likely to participate in online surveys or questionnaires, particularly for topics related to health or social issues. Research suggests that women tend to be more engaged in health-seeking behaviors and more willing to share their experiences. Thirdly, although internet access is becoming more equitable in our country, there might still be subtle gender disparities in internet usage patterns or comfort levels with online platforms, potentially influencing participation rates. Fourth, another limitation is the potential for self-reporting bias, as the data were collected through self-administered questionnaires, potentially leading to an overestimation of positive health practices and an underreporting of less desirable behaviors, such as having multiple sexual partners or age of onset at younger ages. Fifth, a significant proportion of the participants in our study were medical students, and thus the level of STI knowledge might have been underestimated in the general Romanian youth population. In addition, university students may differ from the general population in terms of socioeconomic status, educational attainment, and access to information, potentially influencing their knowledge and attitudes toward sexual health. Sixth, concerning the recruiting strategy, which used mixed methods, it is impossible to completely guarantee that no individual completed the survey multiple times, although several measures were taken to minimize this risk. A significant portion of the responses was collected in a controlled classroom setting under the direct supervision of instructors, and the survey introduction emphasized the importance of honest and single participation. Although the possibility of some duplicate entries cannot be entirely ruled out, it is believed that their number would be minimal and unlikely to significantly impact the overall findings. On the other hand, the recruitment strategy was employed to maximize participation and reach a diverse sample. We acknowledge that our findings may not be directly generalizable to the entire Romanian population.

While acknowledging certain limitations, this study’s high response rate, as well as the large sample size of the respondents and the complexity of the items for evaluating sociodemographic data, yielded important insights into promoting sexual education across one’s lifespan. However, we also emphasize the value of our study in providing insights into the knowledge, attitudes, and behaviors of a specific population group (university students), which can inform targeted interventions and future research.

The following are our suggestions for interventions and future research in order to support factors and resources which could influence health outcomes. Further research is needed to specifically investigate the accuracy of self-perceived STI knowledge across different demographics. This would allow for more targeted and effective interventions to address knowledge gaps and improve sexual health outcomes. Based on these potential trends, targeted interventions could include age-specific education (i.e., developing tailored sexual health education programs for younger age groups while focusing on foundational knowledge and addressing common misconceptions); increasing access to sexual health information and services in rural areas through community-based programs, online resources, and partnerships with local healthcare providers; designing culturally sensitive sexual health interventions addressing potential barriers to accessing information and promoting culturally relevant messaging; and providing continuing education opportunities for individuals, emphasizing the evolving nature of sexual health knowledge and encouraging ongoing learning.

## 5. Conclusions

This study highlighted the critical need for health communication campaigns to enhance STI-related knowledge and vaccine literacy to improve the vaccination rates among young people in Romania. The identified knowledge gaps, frequent misconceptions, and barriers to testing STIs underscore the importance of comprehensive sexual health education, public health initiatives for reducing the stigma associated with STIs, and improved access to healthcare services for young people. Tailored interventions targeting specific demographic and socioeconomic groups are essential to effectively address the complex challenges posed by STIs. By implementing evidence-based strategies informed by these findings, we can move toward a future where STIs are less prevalent and individuals feel empowered to protect their sexual health.

## Figures and Tables

**Table 1:** Sociodemographic characteristics of the responders by number and percentage.

Variables	Categories	No. (Percentage)
Sex	Male	221 (20.3)
	Female	865 (79.4)
	Others	3 (0.3)
Background environment	Rural	210 (19.3)
	Urban	879 (80.7)
Ethnicity	Romanian	1033 (94.9)
	Others	56 (5.1)
Religion	Orthodox	914 (83.9)
	Others	175 (16.1)
Field of study	Health science	825 (75.8)
	Non-health science	264 (24.2)
Parent education	Both secondary and primary education	404 (37.1)
	Only mother, higher education	137 (12.6)
	Only father, higher education	87 (8.0)
	Both higher education	461 (42.3)
Family status	Family (parents)	1058 (97.1)
	Others	31 (2.9)
Household status (current living condition)	Single	229 (21.0)
	Family	301 (27.6)
	Relatives	14 (1.3)
	Partner	194 (17.8)
	Flatmates	131 (12.0)
	Roommates	220 (20.2)
Relationship status	Single	692 (63.5)
	Engaged	397 (36.5)
Educational status	University student	835 (76.7)
	Postgraduate student (other)	254 (23.3)

**Table 2:** Demographic data influencing STI knowledge level.

Variable	Item	Total n (%)	STI Knowledge Level		*p* Value
			Poor to Average	Good to Excellent	
Sex	Female	865 (79.4)	46 (5.4)	819 (94.6)	0.002
	Male	221 (20.3)	20 (10.1)	201 (90.9)	
	Other	3 (0.3)	1 (33.3)	2 (66.7)	
Age (years)	18–20	244 (22.4)	32 (13.1)	212 (86.9)	<0.001
	21–25	716 (65.8)	31 (4.3)	685 (95.7)	
	26–30	103 (9.5)	4 (3.9)	99 (96.1)	
	31–35	25 (2.3)	-	25 (100)	
Background environment	Urban	879 (80.7)	51 (5.8)	828 (94.2)	0.081
	Rural	210 (19.3)	16 (7.6)	194 (92.4)	
Ethnicity	Romanian	1033 (94.9)	62 (6)	971 (94)	0.022
	Hungarian	33 (3)	2 (6)	31 (94)	
	Roma	3 (0.3)	1 (33.3)	2 (66.7)	
	Others	20 (1.8)	2 (10)	18 (90)	
Religion	Orthodox	914 (83.9)	51 (5.6)	863 (94.4)	0.05
	Catholic	56 (5.1)	2 (3.6)	54 (96.4)	
	No religion	42 (3.9)	6 (14.3)	36 (85.7)	
	Other	77 (7.1)	9 (11.7)	68 (88.3)	
Parents education	Both, higher education	461 (42.3)	20 (4.3)	441 (95.7)	<0.001
	Only father, higher education	87 (8)	5 (5.7)	82 (94.3)	
	Only mother, higher education	137 (12.6)	9 (6.6)	128 (93.4)	
	Both, secondary or primary education	404 (37.1)	33 (8.2)	371 (91.8)	
Current educational status of responders	Student	835 (76.7)	51 (6.1)	784 (93.9)	<0.001
	Other	254 (23.3)	16 (6.3)	238 (93.7)	
Field of study	Health sciences	825 (75.7)	25 (3)	800 (97)	<0.001
	Non-health sciences	264 (24.3)	42 (15.9)	222 (84.1)	

*p* < 0.05 was considered statistically significant (chi-squared).

**Table 3:** Sexual history influencing STI knowledge level.

Variable	Item	Total n (%)	STI Knowledge Level (Calculated)		*p* Value
			Poor to Average	Good to Excellent	
Sexual orientation	Heterosexual	959 (89.1)	58 (6)	901 (94)	<0.001
	Other	130 (11.9)	9 (6.9)	121 (93.1)	
Age of first sexual intercourse (years)	<14	11 (1)	0 (0)	11 (100)	<0.001
	14–16	137 (12.6)	11 (8)	126 (92)	
	16–18	350 (32.1)	15 (4.3)	335 (95.8)	
	>18	432 (39.7)	22 (5.1)	410 (95)	
	Never	159 (14.6)	19 (11.9)	140 (88)	
First partner age difference	I have never had a sexual partner	90 (8.2)	32 (2.9)	80 (7.35)	0.01
	Younger	112 (10.3)	20 (1.8)	70 (6.4)	
	No difference	446 (41)	93 (8.5)	353 (32.4)	
	Older	441 (40.5)	79 (7.3)	362 (33.2)	
Recent sexual partners (6 months)	0	293 (26.9)	30 (10.2)	263 (89.8)	<0.001
	1–2	751 (69)	36 (4.8)	721 (95.2)	
	3–5	38 (3.5)	1 (2.8)	37 (97.2)	
Stable sexual partner	Yes	705 (64.7)	34 (4.8)	671 (95.1)	0.004
	No	385 (35.3)	33 (8.6)	350 (91.4)	

*p* < 0.05 was considered statistically significant (chi-squared).

**Table 4:** STI awareness and risk perception associated with STI knowledge level.

Variable	Item	Total n (%)	STI Knowledge Level (Calculated)		*p* Value
			Poor to Average	Good to Excellent	
Self-assessment of STI knowledge	Absent	11 (1)	6 (54.5)	5 (45.5)	<0.001
	Poor	53 (4.9)	11 (20.8)	42 (79.2)	
	Average	270 (24.8)	25 (9.3)	245 (90.7)	
	Good	588 (54)	22 (3.7)	566 (96.3)	
	Excellent	167 (15.4)	3 (1.8)	164 (98.2)	
Perceived value of STI info	Positive perception	1082 (99.4)	63 (5.8)	1019 (94.2)	<0.001
	Negative perception	7 (0.6)	4 (57)	3 (43)	
Risk perception	Low risk	1071 (98.3)	218 (20)	853 (78.3)	0.17
	High risk	18 (1.7)	6 (0.6)	12 (1.1)	
Self-perception of unprotected sex risks	Risk-aware	1066 (97.9)	207 (19)	859 (79)	<0.001
	Risk-unaware	23 (2.1)	17 (1.6)	6 (0.6)	
Preferred sources of information	Health specialists	1060 (97.3)	207 (19)	853 (78.3)	0.93
	Media	611 (56.1)	122 (11.2)	489 (44.9)	
	Community	580 (53.3)	111 (10.2)	469 (43.1)	

*p* < 0.05 was considered statistically significant (chi-squared).

**Table 5:** STI prevention attitudes influencing level of knowledge.

Variable	Item	Total n (%)	STI Knowledge Level (Calculated)		*p* Value
			Poor to Average	Good to Excellent	
HIV testing history	Yes	293 (26.9)	10 (3.4)	282 (96.6)	<0.001
	No	783 (71.9)	56 (7.1)	727 (92.9)	
	I do not know	14 (1.3)	1 (7.1)	13 (92.8)	
Others STI testing history	Yes	387 (35.4)	9 (2.4)	377 (97.6)	<0.001
	No	691 (63.5)	58 (8.4)	633 (91.6)	
	I do not know	12 (1.1)	-	12 (100)	
Preferred methods of contraception	Condom	828 (76)	159 (14.6)	669 (61.4)	<0.001
	Oral contraceptive	152 (14)	30 (2.8)	122 (11.2)	
	Pullout method	279 (25.6)	37 (3.4)	242 (22.2)	
	Other	27 (2.5)	4 (0.4)	23 (2.1)	
	None	100 (9.2)	33 (3)	67 (6.2)	
	Never had sexual contact	105 (9.7)	27 (2.5)	78 (7.2)	
Attitude toward contraceptive methods	I still use contraceptive methods	556 (51.1)	22 (4)	534 (96)	<0.001
	I do not have sexual contact	285 (26.2)	31 (11)	254 (89)	
	Pleasure is greater without contraception	95 (8.7)	5 (5.3)	90 (94.7)	
	I believe that my partner is responsible for contraception	51 (4.7)	3 (5.9)	48 (94.1)	
	I want a child	44 (4)	-	44 (100)	
	I do not know what this entails	23 (2.1)	5 (21.7)	18 (78.3)	
	Contraception is not healthy	11 (1)	1 (10)	10 (90)	
	I do not want to answer	24 (2.2)	-	24 (100)	

*p* < 0.05 was considered statistically significant (chi-squared).

**Table 6:** Multiple regression regarding STI level of knowledge and its influencing factors.

Coefficients								
Model				df		Exp(B)	95% C.I. for EXP(B)	
	B	Std Error	Wald		Sig. *		Lower	Upper
Age	0.673	0.142	22.348	1	<0.001	1.960	1.483	2.591
Gender	0.224	0.216	1.076	1	0.010	1.300	0.819	1.910
Background	−0.100	0.227	0.193	1	0.660	0.905	0.580	1.412
Field of study	−1.695	0.189	80.797	1	<0.001	0.184	0.127	0.266
Ethnicity	−0.285	0.439	0.422	1	0.516	0.752	0.318	1.777
Religion	−0.208	0.266	0.608	1	0.435	0.813	0.482	1.369
Parent education	−0.168	0.070	5.759	1	0.016	0.845	0.737	0.970
Attitude toward contraception	0.171	0.180	0.894	1	0.344	1.186	0.833	1.689
Age of onset of sexual life	0.435	0.184	5.566	1	0.018	1.545	1.076	2.217
Sexual orientation	0.073	0.232	0.098	1	0.754	1.075	0.683	1.693
Constant	1.886	1.200	2.470	1	0.116	6.593		

* *p* < 0.05 was considered statistically significant. C.I. = confidence interval.

## Data Availability

The datasets generated and analyzed during the current study are not publicly available, since they were deliberately collected by the authors for the present study. They may be available from the corresponding author upon reasonable request.

## Supplementary Materials

The following supporting information can be downloaded at https://www.mdpi.com/article/10.3390/vaccines13030322/s1, Supplementary Table S1: Questions and coding for variables included in the analysis.

## Abbreviations

EU	European Union
HIV	Human immunodeficiency virus
HL	Health literacy
HPV	Human papillomavirus
IUD	Intra-uterine device
SHE	Sexual health education
SHK	Sexual health knowledge
STI	Sexually transmitted infection
WHO	World Health Organization
