# Supplementary material for: Beyond Vaccination: Exploring Young Adults’ Awareness, Knowledge, and Attitudes Related to Sexually Transmitted Infections in Romania
Source: Vaccines (Basel). 2025 Mar 18;13(3):322. doi: 10.3390/vaccines13030322 (PMC11945838; doi:10.3390/vaccines13030322)
Supplement: Supplementary file 1 [file vaccines-13-00322-s001.zip › vaccines-3500426-supplementary.pdf]

SUPPLEMENTARY MATERIALS - Questions and coding for variables included in the analysis.

Supplementary Table S1. Questions and coding for variables included in the analysis.

Variables	Questions	Coding
**Sociodemographic**		
Age	How old are you? (Years)	18-35
Sex	What is your sex?	"Female"; "Male"; "Others"
Background	What residential area do you live in?	"Rural"; "Urban"
Ethnicity	What ethnicity are you?	Romanian: "Romanian"; Hungarian/Roma/Other ethnicity: "Other"
Religion	Which religion are you baptized in?	Orthodox: "Orthodox"; Catholic, Protestant, Baptist, no religion: "Others"
Family status	Who were you raised by?	Family (parents): "Parents"; relatives (other than parents)/foster care system/others: "Others"
Housing affiliation (living conditions)	You currently live with:	"Single"; "Family/Relatives"; "Partner"; "Flatmates/Roommates"
**Education and Family Background:**		
Parents education	What is the last school your parents graduated from?	Answered separately for Mother and Father, but the final coding applies to both: primary school (4 grades)/general school (8 grades)/high school studies/vocational school/post-secondary studies: "Both secondary and primary education"; "Only Mother higher education"/"Only Father higher education"; university studies/postgraduate studies: "Both higher education"
Responder's educational status	Are you currently a university student?	Yes: "Student"; No: "Non-student (Other)"
Field of study	What is your field of study?	Medicine/Biological and Biomedical Sciences: "Medical"; Mathematics and Natural Sciences/Engineering sciences/Social sciences/Humanities and arts/Science of sports and physical education/Food industry/Pedagogy/Economics/Informatics/Law: "Non-medical"
**Sexual Health and History**		
Relationship status	Are you currently involved in a relationship?	Yes: "Engaged"; No: "Single"
Age of first sexual intercourse	At what age did you start your sex life?	"Never"; "< 14 years"; "14-16 years"; "16-18 years"; "> 18 years"
First partner age difference	What was the age difference between you and your first sexual partner?	"I've never had a sexual partner"; "Younger"; "No difference"; "Older"
Stable sexual partner	Do you currently have a stable sexual partner?	"Yes"; "No"

Recent sexual partners (last 6 months)	How many sexual partners have you had in the last 6 months?	"0"; "1-2"; "3-5"; "more than 5"
Sexual orientation	What is your sexual orientation?	Heterosexual: "Heterosexual"; Homosexual/Bisexual/Others: "Other"
**STI Awareness**		
Self-assessment of STI knowledge	How do you evaluate the amount of information you have on the subject of sexually transmitted diseases?	Absent/Poor/Average: "Insufficient"; Good/Excellent: "Sufficient"
Perceived value of STI info	Do you consider that having information about sexually transmitted diseases is:	Useful: "Positive perception"; Useless/I don't know: "Negative perception"
Risk perception	How much do you perceive yourself as a person at risk of contracting STIs?	Not at all/moderate risk: "Low risk"; high risk: "High risk"
Self-perception of unprotected sex risks	What is the most important consequence of unprotected sex?	Pregnancy/HIV/Other STI: "Risk-Aware"; I don't know: "Risk-Unaware"
Preferred sources of information	What sources do you consider the most suitable for obtaining information about sexually transmitted diseases?	Doctors/Health specialists: "Health specialists"; Internet/Television/Magazines/Brochures/Encyclopedias/Radio: "Media"; Teachers/Parents/Church: "Community"
**STI Knowledge Assessment**		
Level of knowledge of STI—calculated score [2,31,34,35,36,37,38]	Q19: Which of the following is not a sexually transmitted disease?	Hepatitis A/Pertussis: "Correct answer"; Syphilis/Hepatitis B/Chlamydia/Human Papilloma Virus/TBC/HIV/Gonorrhea: "Incorrect answer"
	Q20: Is there a difference between HIV and AIDS?	Yes, HIV is the infection that causes AIDS syndrome: "Correct answer"; Yes, AIDS is the infection that causes HIV disease/No, there is no difference/I don't know: "Incorrect answer"
	Q21: Which of the following is not a way of transmitting STIs?	Sharing personal items: "Correct answer"; Blood transfusions/Sexual intercourse/Sharing injection needles/From mother to child at birth: "Incorrect answer"
	Q22: Which of the following activities can transmit HIV?	Homosexual intercourse/Heterosexual intercourse: "Correct answer"; Insect bites/Only anal sex/Kissing and touching/I don't know: "Incorrect answer"
	Q23: Can a person contract STDs through oral sexual contact?	Yes/Yes, especially when seminal fluid comes into contact with the mucous membranes: "Correct answer"; No/Maybe, but I'm not sure: "Incorrect answer"
	Q24: An HIV-positive person can transmit the disease if they have had sexual contact with another person:	Once: "Correct answer"; Twice/At least 3 times/Several times, but I'm not sure/I don't know: "Incorrect answer"
	Q27: Can oral contraceptives prevent a sexually transmitted infection?	No: "Correct answer"; Yes/I don't know: "Incorrect answer"
	Q28: For which STIs is there a vaccine?	HPV: "Correct answer"; Chlamydia/HIV/Syphilis/Gonorrhea: "Incorrect answer"
	Q29: Can HPV cause cancer in women and men?	Yes: "Correct answer"; No/I don't know: "Incorrect answer"

	Q31: Considering that they can be cured, how dangerous are STIs?	Dangerous/Very dangerous: "Correct answer"; Not at all/Moderately dangerous: "Incorrect answer"
	Q34: Which of the following do you think are the most effective against STIs?	Condom: "Correct answer"; Oral Contraceptives/interrupted coitus/IUD/diaphragm/cervical cap/sponge/vaginal ring/injections/tubal ligation/vasectomy/hormonal implants/none: "Incorrect answer"
**STI Prevention Attitudes**		
Preferred methods of contraception	What contraceptive methods do you generally use?	"Condom"; "Oral Contraceptives"; "Interrupted coitus"; "IUD"; "Diaphragm"; "Cervical cap"; "Sponge"; "Vaginal ring"; "Injections"; "Tubal ligation"; "Vasectomy"; "Hormonal implants"; "None"
Reason for no contraception	If you are not currently using contraceptive methods, what is the reason?	"I don't know what contraception means"; "Pleasure is greater without contraception"; "I want a child"; "I believe the partner is responsible for contraception"; "Contraception is not healthy"
Contraception testing	Which of the following have you already used?	"Condom"; "Oral Contraceptives"; "Interrupted coitus"; "IUD"; "Diaphragm"; "Cervical cap"; "Sponge"; "Vaginal ring"; "Injections"; "Tubal ligation"; "Vasectomy"; "Hormonal implants"; "None"
HIV testing history	Have you ever had an HIV test?	"Yes"; "No"; "I don't know"
STI testing history	Have you ever been tested for a sexually transmitted disease?	"Yes"; "No"; "I don't know"
